# Non-Traditional Cardiovascular Risk Factors in Adolescents with Obesity and Metabolic Syndrome May Predict Future Cardiovascular Disease

**DOI:** 10.3390/nu15204342

**Published:** 2023-10-12

**Authors:** Athanasia Tragomalou, George Paltoglou, Maria Manou, Ioannis V. Kostopoulos, Sofia Loukopoulou, Maria Binou, Ourania E. Tsitsilonis, Flora Bacopoulou, Penio Kassari, Marina Papadopoulou, George Mastorakos, Evangelia Charmandari

**Affiliations:** 1 Center for the Prevention and Management of Overweight and Obesity, Division of Clinical and Translational Research in Endocrinology, First Department of Pediatrics, National and Kapodistrian University of Athens Medical School, ‘Aghia Sophia’ Children’s Hospital, 11527 Athens, Greece; nansymou@hotmail.com (A.T.); gpaltoglou@gmail.com (G.P.); mariamanou93@hotmail.com (M.M.); mmpinou@hotmail.com (M.B.); peniokassari@gmail.com (P.K.); marinageorpap@gmail.com (M.P.); 2 Division of Endocrinology and Metabolism, Center of Clinical, Experimental Surgery and Translational Research, Biomedical Research Foundation of the Academy of Athens, 11527 Athens, Greece; 3 Flow Cytometry Unit, Section of Animal and Human Physiology, Department of Biology, National and Kapodistrian University of Athens, 15784 Athens, Greece; gikosto@gmail.com (I.V.K.); rtsitsil@biol.uoa.gr (O.E.T.); 4 Department of Pediatric Cardiology, ‘Aghia Sophia’ Children’s Hospital, 11527 Athens, Greece; sofialoukopoulou@hotmail.com; 5 Center for Adolescent Medicine in Adolescent Health Care, First Department of Pediatrics, National and Kapodistrian University of Athens Medical School, ‘Aghia Sophia’ Children’s Hospital, 11527 Athens, Greece; bacopouf@hotmail.com; 6 Division of Endocrinology, Diabetes Mellitus and Metabolism, Second Department of Obstetrics and Gynecology, National and Kapodistrian University of Athens Medical School, ‘Aretaieion’ University Hospital, 11527 Athens, Greece; mastorakg@gmail.com

**Keywords:** obesity, cardiovascular disease, metabolic syndrome, atherosclerosis, cardiovascular risk factors, cytokines, carotid intima-media thickness

## Abstract

Obesity in adolescence is associated with significant morbidity and predisposes adolescents to the development of cardiovascular disease (CVD). Although a number of traditional CVD risk factors have been identified in youth, limited data exist regarding non-traditional CVD risk factors. In 89 adolescents with metabolic syndrome (MetS), with 60 age-, gender-, and BMI-matched controls, we determined the non-traditional CVD risk factors (hs-CRP, TG/HDL ratio, ApoB/ApoA1 ratio, NAFLD) in order to investigate whether they may be used as biomarkers for predicting future CVD, and we evaluated their response to the implementation of a multidisciplinary, personalized, lifestyle intervention program for 1 year. We demonstrated that the TG/HDL ratio, IL-2, IL-6, IL-17A, and INF-γ were significantly increased in subjects with MetS than in controls, and may be used as biomarkers to predict future CVD. Subjects with MetS had an increased mean carotid intima-media thickness (cIMT) and prevalence of NAFLD than the controls, while the prevalence of NAFLD correlated strongly with cIMT and IL-6 concentrations. Most of the non-traditional cardiovascular risk factors improved following the implementation of a lifestyle intervention program. These findings indicate that adolescents with MetS may have a greater risk for developing atherosclerosis early in life, while early lifestyle intervention is crucial for preventing the arteriosclerotic process in youth.

## 1. Introduction

Cardiovascular disease (CVD) is considered to be one of the main causes of mortality worldwide. More than one third of deaths in the “affluent societies” of the Western world are due to CVD [1]. Despite the progress made in its prevention and treatment, recent epidemiological data from the American Heart Association (AHA) demonstrate increased morbidity and mortality from CVD, which imposes a substantial risk to public health and healthcare systems all over the world [1]. The etiology of CVD is multifactorial. A number of traditional cardiovascular risk factors have been associated with CVD, such as obesity, diabetes mellitus type 2 (DM2), hypertension, dyslipidemia, metabolic syndrome (MetS), smoking, and an unhealthy lifestyle [2].

CVD includes a wide range of clinical manifestations, such as myocardial infarction, cardiomyopathy, cardiac dysrhythmias, hypertension, and, mainly, atherosclerosis [3], the cause of vascular endothelium dysfunction. The atherosclerotic process begins early in life, and may occur at the beginning of life without any clinical signs or symptoms. Damage to the aorta and coronary arteries occurs later, usually in late adolescence [3,4]. The impaired function of the adipose tissue, as occurs in obesity, leads to the production and release of pro-inflammatory, atherogenic cytokines, diabetogenic factors, and oxidative stress [5]. Subsequently, cytokines, such as tumor necrosis factor-α (TNF-α) and interleukin-1 (IL-1), are released, the inflammatory cascade is activated, leukocytes are chemotactically attracted, foamy macrophages are generated, and, finally, the stable or unstable atheromatous plaque is created. The expansion of adipose tissue results in endothelial dysfunction, increased fat deposition in the striated muscle of the aorta, an increased thickness of the arterial wall, and abnormalities in the coronary vessels, which appear in early childhood and adolescence [6].

Consequently, obesity—the state of the excessive accumulation of adipose tissue—in childhood and adolescence is considered to be one of the most significant cardiovascular risk factors [7,8], given that it is strongly associated with dyslipidemia, hypertension, insulin resistance, and impaired glucose tolerance. The co-occurrence of these disorders, referred to as metabolic syndrome (MetS), may predict mortality from ischemic heart disease and DM2 [9]. MetS results from a complex interaction of many intrinsic and extrinsic factors. Visceral obesity promotes the onset of MetS, while chronic inflammation and insulin resistance contribute to metabolic dysfunction [10,11].

The definition of MetS remains controversial. According to the International Diabetes Federation (IDF) and the National Heart, Lung, and Blood Institute (NHBLI), for the adult population, MetS is defined when three of the following five risk factors coexist: (1) an increased waist circumference (WC) (with special values and criteria per population and country); (2) triglycerides (TG) ≥ 150 mg/dL; (3) high-density lipoprotein (HDL) < 40 mg/dL in men and <50 mg/dL in women; (4) an increased arterial blood pressure (BP) (systolic blood pressure (SBP) ≥ 130 mmHg or diastolic blood pressure (DBP) ≥ 85 mmHg); and (5) fasting glucose concentrations > 100 mg/dL [12,13]. The risk of atherosclerotic cardiovascular disease, however, is not fully explained by the cluster of cardiovascular risk factors that define MetS, especially for the pediatric population, for which a clear definition of MetS has not yet been established, owing to several difficulties such as normal changes in weight (Wt) and height (Ht) in developing children, different hormonal influences in adolescence, and different balances of the factors that regulate glucose metabolism among children and adolescents with obesity compared to adults.

According to the IDF, children and adolescents aged 10–16 years are diagnosed with MetS if they have visceral obesity (WC > 90th percentile) and the presence of at least two additional adult MetS criteria. In the post-adolescent age of >16 years, the IDF criteria that apply to the diagnosis of MetS in adults are used. Children younger than 6 years are excluded from this definition because there is scant evidence for this clinical group. For children aged 6–10 years, the diagnosis of MetS cannot be defined; however, special attention must be paid to children with visceral obesity and WC > 90th percentile, especially if there is a family history of metabolic and cardiovascular disease [14].

Therefore, given the ambiguity in the definition of MetS in childhood and adolescence, it becomes necessary to investigate the role of novel, non-traditional cardiovascular risk factors, such as high-sensitivity CRP (hs-CRP), TG/HDL ratio, apolipoprotein (Apo) B/A1 ratio, leptin, adiponectin, homocysteine, interleukins (IL-2, IL-4, IL-6, IL-10, IL-17A), TNF-α, and interferon-γ (INF-γ), in predicting future CVD in order to detect it earlier and prevent it. These non-traditional cardiovascular risk factors have been associated with CVD, as well as structural and factional cardiac and vascular changes, independently of the traditional cardiovascular risk factors of MetS [15,16,17,18,19].

## 2. Aim

The aim of our study was to (i) determine non-traditional cardiovascular risk factors in children and adolescents with obesity, (ii) correlate them with the subclinical atheromatous risk, (iii) determine their value in predicting future CVD and their use as biomarkers that may indicate susceptibility to CVD, and (iv) evaluate their response to the implementation of a comprehensive, personalized, lifestyle intervention program of diet, sleep, and exercise for 1 year. The non-traditional cardiovascular risk factors that we studied were the following: hs-CRP, TG/HDL ratio, ApoA1, ApoB, ApoB/ApoA1 ratio, leptin, adiponectin, homocysteine, IL-2, IL-4, IL-6, IL-10, IL-17A, TNF-α and INF-γ, and non-alcoholic fatty liver disease (NAFLD). The above factors have been repeatedly studied in adults; however, limited data exist concerning children and adolescents.

## 3. Subjects and Methods

### 3.1. Study Design

A prospective study was designed from participants attending our Center for the Prevention and Management of Overweight and Obesity in Childhood and Adolescence. The study was carried out at the Division of Clinical and Translational Research in Endocrinology, First Department of Pediatrics, National and Kapodistrian University of Athens Medical School. The patient group included children and adolescents with obesity and MetS, while the control group consisted of age-, gender-, and body mass index (BMI)-matched children and adolescents who had obesity. Both groups participated in a comprehensive, multidisciplinary, personalized lifestyle intervention program of diet, sleep, and exercise for one year [20,21]. Data were collected from the two groups of participants at the beginning of the study and one year after intervention. This study was carried out in accordance with the Helsinki Declaration and received approval from the Ethics Committee of Human Research of the ‘Aghia Sophia’ Children’s Hospital (Approval Number: EB-PASCH-MoM: 28/11/2013, Re: 10290-14/05/2013). Parents and legal guardians were fully informed about the aims of the study, written consent was signed by the parents of all participants, and assent was given by all subjects.

### 3.2. Participants

At initial assessment, all participants attending our Center for the Prevention and Management of Overweight and Obesity in Childhood and Adolescence were classified as having obesity, overweight, or a normal BMI, according to the International Obesity Task Force (IOTF) cut-off points. From a total of one thousand four hundred (*n* = 1400) children and adolescents with obesity, we selected eighty-nine (89), who fulfilled the IDF criteria for MetS. A control group of sixty (*n* = 60) children and adolescents without MetS, matched for age, gender, and BMI, was selected from the cluster of 1400 obese subjects. All participants underwent clinical examination by a pediatrician and a pediatric endocrinologist, were evaluated by a pediatric dietician, a professional fitness personal trainer and—when necessary—a pediatric clinical psychologist, and followed a personalized, integrated, multidisciplinary management lifestyle intervention program, as mentioned previously [20,21,22,23,24].

### 3.3. Study Protocol

#### 3.3.1. Initial Assessment

All participants underwent a detailed clinical examination, including pubertal Tanner staging, and standard anthropometric measurements (Wt, Ht, WC, hip circumference, waist to hip ratio (WHR), waist to height ratio (WHtR)) were obtained by a single, trained observer early in the morning on the day of the first visit, as previously described [21,22,23,24,25]. The BMI was determined as the body Wt (in kg) divided by the Ht (in m) squared and expressed in kg/m^2^, and the z-score BMI was calculated. In addition, the BP was determined using a sphygmomanometer with a suitable cuff for the age of the participant.

Following the initial clinical assessment, a fasting blood sample for hematologic, biochemical, and endocrinologic investigations was obtained at 08:00 h, including an evaluation of the “non-traditional” cardiovascular risk factors described above (hs-CRP, TG/HDL ratio, ApoB/ApoA1 ratio, leptin, adiponectin, homocysteine, IL-2, IL-4, IL-6, IL-10, IL-17A, TNF-α, and INF-γ). Samples were centrifuged and separated immediately after collection, and were stored at –80 °C until assayed. In addition, all subjects had a liver ultrasound scan to determine the presence of NAFLD, as well as undergoing echocardiography and ultrasonography of the carotid arteries to determine the carotid intima-media thickness (cIMT). 

#### 3.3.2. Intervention

Following the initial assessment by the pediatrician and pediatric endocrinologist, all subjects were evaluated by a pediatric dietician, and a 24 h recall of their diet was documented according to the 2010 USDA guidelines [20,21,22,24,26]. More specifically, the pediatric dietitian recorded the number of meals and snacks per day, the usual preferences in terms of food choice, as well as any possible food allergies. Furthermore, information was obtained on how the meals were prepared, how often each food category was offered, and what the food choices were for other family members. In addition, information was obtained on the frequency and quantity of the consumption of fluids (water, milk, juices, and other beverages), as well as unhealthy food (sweets, ice cream, chocolate, biscuits, chips). Furthermore, detailed guidance for proper sleep was given to all subjects depending on their age and according to the American Academy Consensus Guidelines. These recommendations indicated 9 to 12 h of sleep per 24 h for children aged 10–12 years, and 8 to 10 h per 24 h for adolescents aged 13–18 years old [27]. There was also a recommendation for uninterrupted sleep, as early as possible before midnight, and preferably at the same time on a daily basis.

Subsequently, all patients and their parents were informed about obesity and its complications in order to motivate the whole family to adopt a healthy lifestyle; they were guided on changes to their nutritional habits, and a personalized dietetic plan was given according to the guidelines proposed by the National Infant, Child, and Adolescent Nutrition Guide [28]. More specifically, they were advised to eat regularly throughout the day, and have three main meals (breakfast, lunch, and dinner) and two healthy snacks at mid-morning and mid-afternoon. The importance of breakfast consumption was emphasized, and the composition of the main meals was suggested according to “My Plate”, a visualized approach of the 2010 USDA guidelines [20,21,22,23,24,26].

Furthermore, all participants were evaluated by a professional personal trainer, and special instructions were given on the appropriate sports activities according to the child’s interests and skills [20]. The aim was to recommend a personalized physical activity plan, which would be highly enjoyable and entertaining [20,21,22].

Scheduled appointments were arranged every month, according to a standardized follow-up protocol, for all participants prospectively for one year, as previously described [20,21,22,23,24]. Upon each follow-up visit, all participants underwent a detailed clinical examination, and were evaluated by the pediatrician, pediatric endocrinologist, pediatric dietician, and the professional personal trainer, and counseling on lifestyle interventions was provided once again, as described above [29,30].

#### 3.3.3. Annual Assessment

After one year of implementation of the lifestyle intervention program, at the annual assessment, all participants were admitted to the Endocrine Unit early in the morning, and underwent a complete clinical and laboratory evaluation, as at the initial evaluation described above, including hematologic, biochemical, and endocrinologic investigations, the determination of the traditional and non-traditional cardiovascular risk factors, a liver ultrasound scan, an echocardiogram, and a carotid artery ultrasound scan. 

### 3.4. Assays

The ADVIA 2110i analyzer (Roche Diagnostics GmbH, Mannheim, Germany) was used for the determination of all hematologic investigations. The concentrations of glucose, total cholesterol, TG, and HDL-cholesterol were measured using the ADVIA 1800 Siemens analyzer (Siemens Healthcare Diagnostics, Tarrytown, NY, USA). ApoA1, ApoB and lipoprotein (a) concentrations were determined via the means of latex particle-enhanced immunonephelometric assays on the BN ProSpec nephelometer (Dade Behring, Siemens Healthcare Diagnostics, Liederbach, Germany). Hemoglobin A1C (HbA1C) was determined using an automated glycohemoglobin analyzer HA-8160 (Arkray, Kyoto, Japan). An automated electrochemiluminescence immunoassay analyzer (Cobas e411, Roche Diagnostics GmbH, Mannheim, Germany) was used to determine follicle-stimulating hormone (FSH), luteinizing hormone (LH), estradiol, ferritin, and insulin concentrations. Thyroid-stimulating hormone (TSH), free thyroxine, anti-thyroid peroxidase antibodies (Anti-TPO), anti-thyroglobulin antibodies (Anti-TG), cortisol, androstenedione, testosterone, dehydroepiandrosterone sulfate (DHEAS), insulin-like growth factor-I (IGF-I), and hs-CRP concentrations were measured via chemiluminescence immunoassays on an IMMULITE 2000 immunoassay system (Siemens Healthcare Diagnostics Products Ltd., Camberley, Surrey, UK). 

Leptin was determined using a Milliplex Map Human Metabolic Panel (Millipore Corp, St. Charles, IL, USA), which is based on the Luminex xMAP technology. Adiponectin was assayed using an enzyme-linked immunosorbent assay (ELISA) method (Orgenium laboratories, Helsinki, Finland). The cytokines IL-2, IL-4, IL-6, IL-10, IL-17A, TNF-α, and INF-γ were determined using the Cytometric Bead Array (CBA) Human Th1/Th2/Th17 kit (BD Biosciences, San Jose, CA, USA) [31].

### 3.5. Echocardiography

Using echocardiography, the common carotid intima-media thickness (cIMT) of the right and left common carotid arteries was determined in the final diastolic phase with the patient in the supine position, with the neck slightly extended and turned 45° to the opposite side of the examined side, by using a three-electrode electrocardiogram (monitoring the end of the R wave). The examination was performed on both carotid arteries (right and left, RCCA medial wall thickness of the right internal carotid artery, LCCA medial wall thickness of the left internal carotid artery) by the same trained pediatric cardiologist (SL). The carotid wall consists of three parts, as depicted in Triplex: two light lines separated by a darker area (the lumen-intima and the media-adventitia interface). The cIMT was defined as the distance between these two light lines. A 2D-echocardiogram was used in order to measure the ventricular dimensions using echo parameters (the indicators of left ventricular hypertrophy): the intraventricular septal end systole (IVSs) and especially the intraventricular septal end diastole (IVSd), the left ventricular internal diameter end diastole (LVIDd), and the left ventricular internal diameter end systole (LVIDs).

### 3.6. Statistical Analysis

All variables were normally distributed. The results are reported as the mean ± SE. Significant main effects were revealed in the LSD post hoc test. Statistical significance was set at (*p* < 0.05), while strong significance (*p* < 0.01) is also noted. All variables that were assessed at initial assessment and at the annual assessment were compared by employing a repeated measures analysis of variance test (ANOVA). Significant main effects were revealed via Fischer’s (LSD) post hoc test. Insulin resistance was calculated using the homeostasis model assessment (HOMA) method as follows: HOMA-IR = (fasting glucose [mg/dL] × fasting insulin [mU/L])/405. The sample size calculation was performed using the GPower 3.1.9.2 (Universität Kiel, Kiel, Germany) software, and the calculated minimum sample size for the repeated measures ANOVA for an effect size of 0.25 and 0.95 power was 54 participants. Our sample size of 149 participants achieved a post hoc-computed achieved power of 0.99.

Standard forward stepwise multiple linear regression models were employed to investigate potential predictors of the cIMT at times (t) 0′ and 12′ (mean), all taken separately as dependent variables. The association of an increased waist circumference with cardiovascular risk is well known. Visceral obesity initiates the onset of MS, while chronic inflammation and insulin resistance contribute to metabolic dysfunction [15]. When insulin resistance occurs, energy homeostasis is altered, resulting in inappropriate hepatic glucose production and triglyceride accumulation, further resulting in microvascular dysfunction and accelerated coronary atherosclerosis. Based on the above pathophysiological mechanisms, four models were formulated to study the parameters involved in the inflammation that takes place in the adipose tissue [32].

In the first model, the anthropometric parameters (Wt, Ht, BMI, waist and hip circumference, WHR, and WHtR) at initial assessment were taken as independent variables. In the second model, the metabolic syndrome parameters (glucose concentration, SBP, WC, TG, and HDL) at initial assessment were taken as independent variables. In the third model, the glucose metabolism and insulin sensitivity parameters (glucose, insulin, HbA1C, and HOMA-IR) at initial assessment were taken as independent variables. In the fourth model, the adiposity parameters (adiponectin and leptin concentrations, WC, WHtR, and IL-6) at initial assessment were taken as independent variables.

All statistical analyses were performed using the Statistica 8 software (StatSoft, Tulsa, OK, USA).

## 4. Results

As we have mentioned above, eighty-nine (89) children and adolescents with obesity who fulfilled the IDF criteria for MetS were selected from a total of one thousand four hundred (*n* = 1400) obese patients attending our Center for the Prevention and Management of Overweight and Obesity in Childhood and Adolescence (mean age ± SD: 13.19 ± 0.20 years; 59 males (66.3%) and 30 females (33.7%); 15 prepubertal (16.8%) and 74 pubertal (83.1%)). A control group of sixty (*n* = 60) children and adolescents without MetS, matched for age, gender, and BMI (mean age ± SD: 12.05 ± 0.28 years; 37 males (61.7%) and 23 females (38.3%); 17 prepubertal (28.3%) and 43 pubertal (71.7%)) was selected from the cluster of 1400 obese subjects. In total, 149 participants (84 male (56.4%), 65 female (43.6%)) were studied prospectively for one year. 

**Figure 1:**
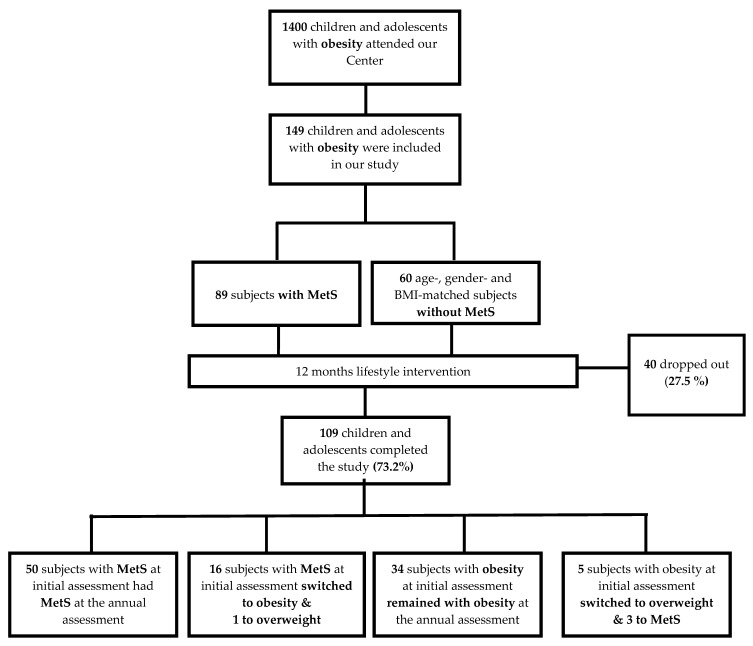
Recruitment and enrollment at baseline and follow-up.

From the total of 149 patients, 40 obese children and adolescents (22 with MetS and 18 controls) did not complete the study (27.5% drop-out rate). The remaining 109 patients completed the study and attended all follow-up appointments for detailed clinical, laboratory, and ultrasound assessments (rate: 73.2%). Sixteen (*n* = 16) patients with MetS at initial assessment demonstrated a decrease in their BMI and an improvement in the rest of the parameters that constitute MetS at the end of the study, and switched to the control group with obesity but no MetS (18%). Furthermore, five patients with obesity and no MetS (control group) at initial assessment became overweight (8.3%) at the end of the study (Figure 1).

The clinical characteristics, anthropometric parameters, hematologic, biochemical, and endocrinologic investigations, traditional and non-traditional cardiovascular risk factors, and echocardiography and ultrasonography findings in all subjects, both at initial and at the annual assessment, are shown in Table 1, Table 2, Table 3 and Table 4. Subjects were classified as having obesity without MetS and obesity with MetS according to the evaluation findings at the initial assessment. 

### 4.1. Clinical Characteristics, Anthropometric Parameters, and Hematologic, Biochemical, and Endocrinologic Investigations

Subjects with MetS had significantly greater Wt, BMI, WC, hip circumference, WHR, and WHtR than subjects without MetS, both at initial and at the annual assessment (*p* < 0.01), while a significant reduction in the BMI, WHR, and WHtR (*p* < 0.01) was observed following the implementation of the 1-year lifestyle intervention program (Table 1A). No significant differences in the hematologic, biochemical, and endocrinologic parameters were noted between the two groups at the initial and at the annual assessment (Table 1B–D).

### 4.2. Traditional Cardiovascular Risk Factors

Subjects with MetS had significantly greater SBP and DBP than those without MetS, both at initial and annual assessment (*p* < 0.01), while a significant reduction in both SBP and DBP (*p* < 0.01) was observed following the implementation of the lifestyle intervention program in the MetS group (Table 2). With respect to the lipid profile, subjects with MetS had significantly lower HDL concentrations than those without MetS, and a significant increase in HDL was observed following the lifestyle intervention in subjects without MetS (*p* < 0.05). The triglyceride concentrations were significantly higher in the MetS subjects at initial assessment, while the ApoA1 concentrations were significantly lower in the MetS group than in the non-MetS group, both at initial and annual assessment.

The insulin concentrations and the HOMA-IR index were significantly higher in the MetS group than the control group, both at initial and annual assessment (*p* < 0.01). No significant difference was noted in the glucose concentrations between the two groups. The total cholesterol, HbA1C, and lipoprotein also showed no significant difference between the two groups.

### 4.3. Non-Traditional Cardiovascular Risk Factors

**Figure 2:**
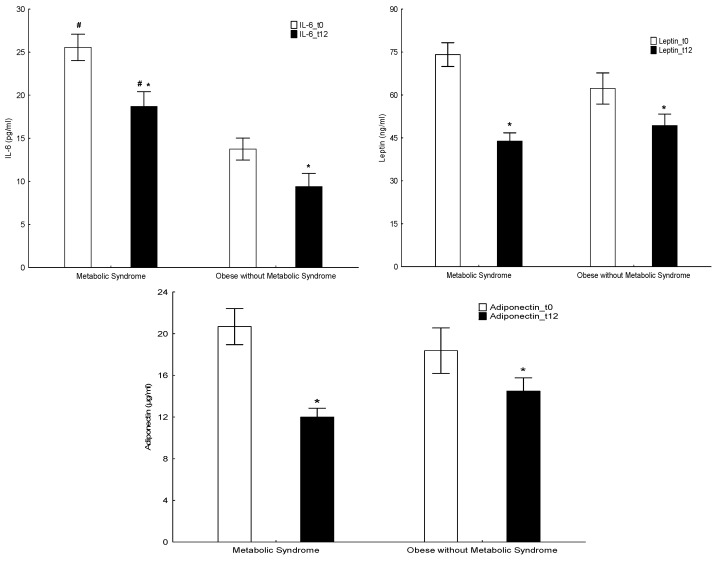
IL-6, leptin, and adiponectin concentrations in the subjects with MetS and without MetS at baseline (t0, white bars) and after intervention (t12, black bars). * indicates a statistically significant difference between the initial and annual assessment time-points; # indicates a statistically significant difference between the two groups at the same time-point.

Subjects with MetS had a significantly greater TG/HDL ratio and IL-6 concentrations than those without MetS, both at initial and annual assessment (*p* < 0.01), while a significant reduction in both parameters was observed following the implementation of the lifestyle intervention program for one year (Table 3; Figure 2). The IL-2, IL-17A, and INF-γ were significantly greater in the MetS group at initial assessment, while the IL-10, IL-17A, TNF-α, and INF-γ were significantly greater in the MetS group at the annual assessment, with no changes observed following the one-year intervention. There were no differences in the leptin and adiponectin concentrations between the two groups at initial and annual assessment; however, there was a significant reduction in the leptin concentrations and a significant increase in the adiponectin concentrations in both groups of participants following the implementation of the lifestyle intervention program, which is consistent with the reduction in adipose inflammation (*p* < 0.01) (Table 3; Figure 2). Although there was no difference in the ApoB/ApoA1 ratio between the two groups at both assessments, a significant reduction in this ratio was noted in the MetS group following the implementation of the lifestyle intervention program, which is also consistent with a decrease in the inflammatory process (*p* < 0.01) (Table 3). Finally, there was no significant difference in the hs-CRP and homocysteine concentrations between the two groups.

### 4.4. Echocardiography and Ultrasonography

**Figure 3:**
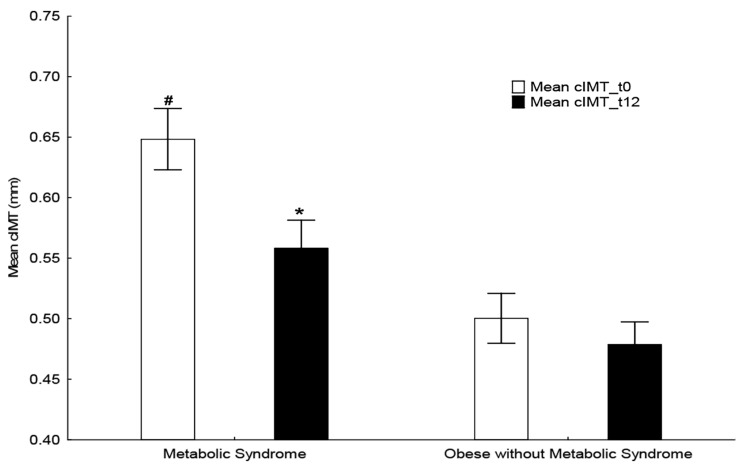
Carotid intima-media thickness (cIMT) in the subjects with MetS and without MetS at baseline and after intervention. * indicates a significant difference between initial and annual assessment time-points; # indicates a significant difference between the two groups at the same time.

Cardiac imaging studies in our population showed that the MetS group had significantly increased dimensions of the left intraventricular septal end systole (IVSs), both at initial and annual assessment, compared with the non-MetS group. Furthermore, the carotid intima-media thickness (cIMT, mean, right carotid artery and left carotid artery) was also significantly increased in the MetS group at initial assessment and was decreased significantly following 1-year of lifestyle intervention (*p* < 0.01) (Table 4) (Figure 3).

Furthermore, a higher percentage of subjects with MetS had NAFLD than those without MetS (51.5% vs. 25.35%, *p* = 0.07), with improvements following the intervention (48.8% vs. 25%) (Table 5). The improvement in NAFLD following the one-year lifestyle intervention program was strongly correlated with the cIMT and IL-6 concentrations, but not with the HOMA-IR index (Table 6). 

### 4.5. Predictors of Carotid Intima-Media Thickness (Table 7)

When the anthropometric parameters (Wt, Ht, BMI, waist and hip circumference, WHR, WHtR) at initial assessment were taken as independent variables in a standard forward stepwise regression model, the Ht at initial assessment was the best positive predictor of cIMTt_0_ (b = 0.284) and cIMTt_12_ (b = 0.271).

When the MetS parameters at initial assessment (SBP, WC, glucose concentration, TG, HDL) were taken as independent variables in a standard forward stepwise regression model, no significant predictors of cIMT were found.

When the glucose metabolism and insulin sensitivity parameters (glucose, insulin, HbA1C, HOMA-IR) at initial assessment were taken as independent variables in a standard forward stepwise regression model, the HOMA-IR was the best positive predictor at initial assessment of cIMTt_0_ (b = 0.365).

When the adiposity parameters (adiponectin and leptin concentrations, WC, WHtR, IL-6) at initial assessment were taken as independent variables in a standard forward stepwise regression model, the IL-6 at initial assessment was the best positive predictor of cIMTt_0_ (b = 0.254) and the WC was the best positive predictor of cIMTt_12_ (b = 0.441).

### 4.6. Limitations

There are some limiting factors in the present study. Firstly, MetS is a relatively rare disease in the pediatric population, for which a clear definition has not yet been formulated in relation to obesity. Given the difficulty in screening patients who meet the MetS criteria, we ended up with a representative sample size, yet capable of revealing statistically significant information. Furthermore, we excluded from this sample patients who had other underlying disease, in order to study the effects of adiposity on inflammation parameters/markers. 

## 5. Discussion

In our study, we determined the non-traditional cardiovascular risk factors in children and adolescents with obesity and MetS, we correlated them with other cardiometabolic risk factors, and we evaluated their response to the implementation of a comprehensive, personalized, multi-disciplinary lifestyle intervention program of diet, sleep, and exercise for 1 year. We demonstrated that the TG/HDL ratio, IL-2, IL-6, IL-17A, and INF-γ were significantly greater in subjects with MetS than in those without MetS, and may therefore be used as biomarkers to predict future cardiovascular disease. We also demonstrated that most of the non-traditional cardiovascular risk factors improved following the implementation of a lifestyle intervention program of a healthy diet, sleep, and exercise for one year.

Regarding the traditional cardiovascular risk factors, children and adolescents with MetS had a significantly greater SBP and DBP than those without MetS, both at initial and annual assessment. These findings concur with previous studies and highlight the role of hypertension as one of the most important traditional modifiable CVD risk factors. High arterial BP is associated with increased afterload and may contribute to abnormal left ventricular hypertrophy in both adults and children with obesity [33]. In addition, we observed a significant reduction in SBP and DBP in children with MetS following the intervention and the adoption of a healthier lifestyle, which demonstrates the value of nutrition and exercise in the prevention of CVD [20,21,22,23,24]. Similar findings were noted in the lipid profile. Dyslipidemia is a major cardiometabolic risk factor, and determining the concentrations of HDL- and LDL-cholesterol is an easy way to assess the atherosclerotic risk. In our study, subjects with MetS had significantly higher TG and lower HDL and ApoA1 concentrations than the control group. 

Regarding the non-traditional cardiovascular risk factors, research studies in adults have demonstrated that the TG/HDL ratio may be used as a sensitive indicator of CVD risk, given that it is related to insulin resistance (Grover’s model) [34,35]. In children, a strong correlation between the TG/HDL ratio with the flow-mediated dilatation (FMD) index was noted, which indicates early onset atherosclerosis [36] and demonstrates that the TG/HDL ratio may be useful as a biomarker that predicts future cardiovascular events.

Another useful indicator of CVD risk, which is not widely used, is the ApoB /ApoA1 ratio, which reflects the balance between atherogenic ApoB and antiatherogenic ApoA1 cholesterol particles and is strongly and positively associated with cardiovascular risk in adults [16]. In one of the few studies carried out on pediatric subjects, there was a strong correlation between an increased ApoB/ApoA1 ratio and increased WC, BMI, fat percentage, DBP, and incidence of MetS [37]. In the present study, we found an increased, although non-statistically significant, ApoB/ApoA1 ratio in the MetS population, which is consistent with the inflammatory response of adipose tissue and the accumulation of atherogenic factors, such as ApoB. After one year of lifestyle interventions, we showed a statistically significant reduction in this ratio in the MetS population, indicating the importance of early intervention in reversing cardiometabolic risk factors. 

Cytokines play an important role in the pathogenesis of MetS. Adiponectin, an anti-inflammatory, anti-atherosclerotic, and anti-oxidative cytokine, and leptin, an inflammatory and pro-oxidative cytokine, were estimated. Adiponectin is a collagen-like protein that improves insulin sensitivity in peripheral tissues [38] by suppressing the inflammatory TNF-α and IL-6 and by reducing the pro-atherogenic LDL and TG concentrations [39,40]. Increased plasma adiponectin concentrations are associated with a lower risk of myocardial infarction (MI) in humans [41], whereas elevated adiponectin concentrations after the onset of MI are positively associated with myocardial salvage and recovery of the ejection fraction [18]. A recent study in pediatric subjects showed that adiponectin concentrations were significantly lower in children and adolescents with MetS than in patients without MetS [42]. In our study, there was no significant difference in the adiponectin concentrations between the two groups, both at initial and annual assessment. 

Leptin concentrations also showed no significant difference between the two groups, both at initial and annual assessment; however, both groups demonstrated a significant decrease in leptin concentrations following intervention, which is consistent with the reduction of inflammation and of CVD risk. Hyperleptinemia and leptin resistance are closely associated with pathologic conditions, such as obesity and DM2, due to leptin resistance in the obesogenic phenotype, whereas lower circulating leptin concentrations are positively correlated with improved insulin sensitivity, and reduced adiposity and inflammation [43,44]. Leptin may be used as a biomarker of CVD risk in preadolescent children; more specifically, leptin concentrations > 13.4 ng/dL are significantly associated with MetS, while for every 1 ng/dL increase in leptin concentration, the odds of developing MetS and its complications increase by 3% [45,46].

The importance of increased homocysteine concentrations for cardiovascular risk in adults has already been demonstrated; however, there are few data for the pediatric and adolescent population. A study in adolescents with MetS showed elevated plasma homocysteine concentrations up to 11.8 ± 5.0 μmol/L, but no significant association with the individual parameters of MetS [47]. In our study, homocysteine concentrations were in the upper normal range according to the adult reference values for both the MetS group and the control group (10.66 ± 0.31 μmol/L vs. 9.90 ± 0.38 μmol/L, respectively) at initial evaluation, with no significant differences between the two groups at both times of assessment.

Concerning other inflammatory markers, hs-CRP and a range of interleukins were estimated at baseline and after one year of intervention. Hs-CRP accurately detects low concentrations of CRP and chronic mild inflammation, and may predict a person’s risk for developing CVD. Accordingly, hs-CRP concentrations of <1 mg/L, 1–3 mg/L, and >3 mg/L separate adults, respectively, into low, intermediate, and high risk for developing CVD (American Heart Association, 2003). A study carried out in children with an increased BMI demonstrated that children with MetS had increased hs-CRP, and showed a positive correlation with BMI, insulin, and TG and a negative correlation with HDL [48]. Therefore, it could be used as a good and sensitive biomarker of cardiovascular risk [49]. In our study, although the mean concentration of hs-CRP was higher in the MetS group, where the inflammation was also increased, no statistically significant difference was noted between the two groups at either time of evaluation.

We also demonstrated that subjects with MetS had significantly increased IL-6 concentrations than those without MetS, both at initial and annual assessment (*p* < 0.01), while a significant reduction in IL-6 was observed following the implementation of the lifestyle intervention program. It is well-known that IL-6 is a mediator of the inflammatory response. Studies carried out in adults with obesity, hyperglycemia, and insulin resistance demonstrate parallel increases in their IL-6 and CRP concentrations with increasing insulin resistance [19]. In children, IL-6 concentrations were also higher in those with obesity than in those with a normal BMI, especially in preadolescence [50]. Our findings concur with these studies, and further indicate that the concentration of IL-6 may be used as a biomarker for predicting cIMT thickness and future atherosclerotic cardiovascular disease.

A significant difference was also observed in the IL-17A, TNF-α, and INF-γ concentrations between the two groups of patients, with the MetS group showing significantly increased concentrations of these pro-inflammatory cytokines than the control group. Once again, these results agree with previous studies demonstrating that adipocyte hypertrophy, hyperplasia, and hypoxia induce adipocyte necrosis, and result in a pro-inflammatory state and the release of the pro-inflammatory cytokines IL-6, IL-1β, TNF-α, and INF-γ [51].

The cardiac imaging studies showed that the average value of the thickness of the mean cIMT in the MetS group was 0.65 ± 0.03 mm at the initial assessment, a value much higher than the normal for that age (normal range: 0.49 ± 0.03 mm) [52,53], and significantly higher than that of the control group (0.50 ± 0.02 mm, *p* < 0.01). In addition, a significant improvement in cIMT was observed in the MetS group after one year of intervention (*p* < 0.01). Furthermore, the thickness of the left ventricular septum during systole (IVSs) was significantly higher in the MetS group than in the control group, both at initial and annual assessment, further underscoring the possible risk of an incipient left ventricular hypertrophy in children with MetS.

Finally, patients with MetS had NAFLD in a greater proportion than the control group. Our findings concur with studies in adults that demonstrate a strong correlation between NAFLD and the components of MetS, mostly insulin resistance and DM2 [54], as well as studies on children that show a significant association with atherosclerosis [55]. We have also demonstrated that, after the appropriate intervention and the implementation of a healthier lifestyle, a parallel improvement was observed in NAFLD, cIMT, and IL-6 concentrations. 

## 6. Conclusions

We demonstrated that the non-traditional cardiovascular risk factors, the TG/HDL ratio, IL-2, IL-6, IL-17A, and INF-γ, were significantly increased in children with MetS than those without MetS, and may therefore be used as biomarkers to predict future cardiovascular disease. Subjects with MetS had increased mean carotid intima-media thicknesses (cIMT) and an increased prevalence of NAFLD than the controls, while the presence of NAFLD correlated strongly with cIMT and IL-6 concentrations. We also demonstrate that most of these risk factors improved following the implementation of a comprehensive, personalized, multi-disciplinary lifestyle intervention program of diet, sleep, and exercise for 1 year. These findings indicate that children with MetS might have a greater risk for developing atherosclerosis early in life, and that early lifestyle intervention is crucial for preventing the arteriosclerotic process in youth.

## Figures and Tables

**Table 1:** Clinical characteristics and anthropometric parameters (A), and hematologic (B), biochemical (C), and endocrinologic investigations (D) in all subjects at initial and annual assessments.

**A. Anthropometry**	**Initial Assessment ** **(*n* = 149)**			**Annual Assessment** **(*n* = 109)**			**P_Between Time-Points_**
	**Obesity ** **without MetS**	**Obesity ** **with MetS**	**P_within Baseline_**	**Obesity without MetS at Initial Assessment **	**Obesity with MetS at Initial Assessment **	**P_within Follow-Up_**	
Age (years)	12.05 ± 0.28	13.19 ± 0.20	NS	13.39 ± 0.33 *	14.30± 0.23 *	NS	<0.01/<0.01
Body weight (kg)	77.13 ± 1.77	96.50 ± 1.98	<0.01	83.15 ± 2.20 *	100.81 ± 2.10 *	<0.01	<0.01/<0.01
Height (cm)	155.57 ± 1.25	163.74 ± 1.12	<0.01	161.40 ± 1.36 *	167.61 ± 1.22 *	<0.01	<0.01/<0.01
BMI (kg/m^2^)	31.65 ± 0.43	35.32 ± 0.62	<0.01	31.77 ± 0.58	35.62 ± 0.53 *	<0.01	NS/<0.05
Waist (cm)	93.56 ± 0.9	107.15 ± 1.35	<0.01	92.82 ± 1.71	107.22 ± 1.48	<0.01	NS/NS
Hip (cm)	103.84 ± 1.27	113.19 ± 1.00	<0.01	106.10 ± 1.86	115.41 ± 1.14 *	<0.01	NS/<0.01
Waist to Hip ratio (WHR)	0.90 ± 0.01	0.95 ± 0.01	<0.01	0.88 ± 0.01 *	0.93 ± 0.01 *	<0.01	<0.01/<0.01
Waist to Height ratio (WHtR)	0.60 ± 0.01	0.65 ± 0.01	<0.01	0.58 ± 0.01 *	0.64 ± 0.01	<0.01	<0.05/NS
**B. Hematology**	**Initial Assessment** **(*n* = 149)**			**Annual Assessment** **(*n* = 109)**			**P_Between time-points_**
	**Obesity ** **without MetS**	**Obesity with MetS**	**P_within baseline_**	**Obesity** **without MetS at Initial Assessment **	**Obesity with MetS at Initial Assessment **	**P_within follow-up_**	
White Blood Cells (WBC) × 10^3^/μL	7.98 ± 0.27	8.09 ± 0.21	NS	8.18 ± 0.29	7.90 ± 0.24	NS	NS/NS
Red Blood Cells (RBC) × 100^3^/μL	4.98 ± 0.06	5.09 ± 0.05	NS	4.98 ± 0.07	5.22 ± 0.17	NS	NS/NS
Hemoglobin (Hb) g/dL	13.07 ± 0.12	13.10 ± 0.13	NS	13.08 ± 0.15	13.75 ± 0.52	NS	NS/NS
Hematocrit (Hct)%	40.61 ± 0.34	40.63 ± 0.37	NS	41.00 ± 0.48	42.11 ± 0.81 *	NS	NS/<0.05
Platelets (PLT) × 10^3^/μL	306.29 ± 9.10	336.18 ± 28.52	NS	309.46 ± 13.59	299.06 ± 9.13	NS	NS/NS
**C. Biochemistry**	**Initial Assessment** **(*n* = 149)**			**Annual Assessment** **(*n* = 109)**			**P_Between time-points_**
	**Obesity** **without MetS**	**Obesity with MetS**	**P_within baseline_**	**Obesity without** **MetS at Initial Assessment **	**Obesity with MetS** **at Initial Assessment **	**P_within follow-up_**	
Urea (mg/dL)	27.32 ± 0.77	26.34 ± 0.70	NS	26.99 ± 1.06	27.02 ± 0.69	NS	NS/NS
Creatinine (mg/dL)	0.57 ± 0.01	0.81 ± 0.20	NS	0.64 ± 0.02 *	0.68 ± 0.02 *	NS	<0.01/<0.01
Uric Acid (mg/dL)	5.22 ± 0.14	5.62 ± 0.12	NS	5.45 ± 0.19	5.82 ± 0.15	NS	NS/NS
Potassium (K) (mmol/L)	4.41 ± 0.07	4.39 ± 0.03	NS	4.60 ± 0.06 *	4.59 ± 0.04 *	NS	<0.01/<0.01
Sodium (Na) (mmol/L)	140.39 ± 0.18	140.65 ± 0.17	NS	140.28 ± 0.27	140.61 ± 0.19	NS	NS/NS
Aspartate Transaminase (AST) (U/L)	20.54 ± 1.16	21.17 ± 0.74	NS	19.26 ± 0.69	21.55 ± 1.28	NS	NS/NS
Alanine Transaminase (ALT) (U/L)	23.59 ± 2.20	28.82 ± 2.05	NS	20.50 ± 1.55	29.33 ± 3.72	<0.05	NS/NS
γ- aminobutyrate Transaminase (γ-GT) (U/L)	14.81 ± 0.69	20.22 ± 2.29	NS	15.42 ± 1.16	18.85 ± 1.43	NS	NS/NS
Albumin (g/dL)	4.67 ± 0.05	4.61 ± 0.03	NS	4.61 ± 0.03	4.67 ± 0.03	NS	NS/NS
Alkaline Phosphatase (U/L)	212.36 ± 11.71	200.62 ± 9.45	NS	174.95 ± 14.86 *	178.56 ± 10.38 *	NS	<0.01/<0.01
Phosphate (mg/dL)	4.66 ± 0.07	4.53 ± 0.08	NS	4.55 ± 0.09	0.49 ± 0.07	NS	NS/NS
Calcium (Ca) (mg/dL)	9.79 ± 0.04	9.77 ± 0.03	NS	9.78 ± 0.05	9.75 ± 0.03	NS	NS/NS
Ferritin (μg/L)	51.37 ± 3.83	59.75 ± 4.52	NS	40.53 ± 4.05	61.30 ± 6.81	<0.05	NS/NS
**D. Endocrinology**	**Initial Assessment** **(*n* = 149)**			**Annual Assessment** **(*n* = 109)**			**P_Between time-points_**
	**Obesity ** **without MetS**	**Obesity with MetS**	**P_within baseline_**	**Obesity without ** **MetS at Initial Assessment **	**Obesity with MetS at Initial Assessment **	**P_within follow-up_**	
Thyroid stimulating hormone (TSH) (μUI/mL)	3.48 ± 0.20	3.42 ± 0.019	NS	2.83 ± 0.19 *	3.22 ± 0.27	NS	<0.05/NS
Free thyroxine (FT4) (ng/dL)	1.12 ± 0.02	1.07 ± 0.02	NS	1.30 ± 0.02	1.28 ± 0.03	NS	<0.01/<0.01
Triiodothyronine (T3) (ng/dL)	141.27 ± 3.89	139.33 ± 3.06	NS	124.92 ± 4.18	123.88 ± 2.97	NS	<0.01/<0.01
Anti-TG (IU/mL)	20.53 ± 0.44	48.32 ± 14.25	NS	18.44 ± 1.09	26.72 ± 6.85	NS	NS/NS
Anti-TPO (IU/mL)	43.76 ± 21.17	43.10 ± 16.11	NS	13.22 ± 1.42	25.60 ± 7.31	NS	NS/NS
Insulin-like growth factor I (IGF-I) (ng/mL)	268.97 ± 15.61	273.50 ± 11.68	NS	295.65 ± 35.09	312.02 ± 18.09 *	NS	NS/<0.01
Prolactin (PRL) (ng/mL)	11.97 ± 0.83	11.73 ± 0.55	NS	10.74 ± 0.75	11.31 ± 0.70	NS	NS/NS
Luteinizing hormone (LH) (mUI/mL)	3.39 ± 0.59	8.57 ± 4.79	NS	5.56 ± 1.10	3.68 ± 0.28	NS	NS/NS
Follicle-stimulating hormone (FSH) (mUI/mL)	3.16 ± 0.38	3.35 ± 0.23	NS	4.13 ± 0.47	3.51 ± 0.25	NS	<0.05/NS
Estradiol (pg/mL)	27.67 ± 5.48	23.10 ± 3.47	NS	39.18 ± 5.95	35.21 ± 3.08	NS	NS/<0.05
Testosterone (ng/mL)	64.83 ± 15.09	97.71 ± 12.63	NS	177.21 ± 23.15	176.43 ± 17.33	NS	<0.01/<0.01
Cortisol (μg/dL)	12.85 ± 0.72	12.55 ± 0.54	NS	13.96 ± 0.92	13.42 ± 0.75	NS	NS/NS
Androstenedione (ng/dL)	1.25 ± 0.13	1.67 ± 0.25	NS	1.76 ± 0.20	1.83 ± 0.16	NS	NS/NS
Dehydroepiandrosterone (μg/dL)	142.03 ± 12.82	171.44 ± 10.76	NS	193.04 ± 22.17	246.44 ± 29.72	NS	<0.01/<0.01
Parathyroid hormone (PTH) (pg/dL)	40.78 ± 2.08	43.51 ± 2.07	NS	28.08 ± 1.63	45.36 ± 21.26	NS	NS/NS
Vitamin D (25-OHVitD) (ng/mL)	21.28 ± 0.64	19.48 ± 1.04	NS	27.25 ± 1.36	21.81 ± 1.19	NS	NS/NS

All results are presented as mean ± SE. Subjects were classified as having obesity according to IOTF criteria and as having obesity with MetS according to IDF criteria at initial assessment. The table presents the comparisons between the two groups at both initial and annual assessment. All measured variables were compared by employing repeated measures ANOVA. Significant main effects were revealed in the LSD post hoc test. Statistical significance was set at (*p* < 0.05), while strong significance (*p* < 0.01) is also noted. NS: non-significant (*p* > 0.05) difference. * indicates significant difference between initial and annual assessment time-points, respectively. The *p*-values between the two time-points refer to subjects with obesity and MetS and obesity without MetS.

**Table 2:** Traditional cardiovascular risk factors, in all subjects at initial and annual assessments.

Traditional CVD Risk Factors	Initial Assessment (*n* = 149)			Annual Assessment (*n* = 109)			P_Between Time-Points_
	Obesity without MetS	Obesity with MetS	P_within Baseline_	Obesity without MetS at Initial Assessment	Obesity with MetS at Initial Assessment	P_within Follow-Up_	
Systolic Blood Pressure (SBP, mmHg)	114.30 ± 1.09	127.04 ± 1.28	<0.01	114.46 ± 1.45	122.07 ± 1.2 *	<0.01	NS/<0.01
Diastolic Blood Pressure (DBP, mmHg)	68.35 ± 1.13	74.91 ± 1.02	<0.01	74.55 ± 1.44 *	78.30 ± 1.23 *	<0.01	<0.01/<0.01
Cholesterol (mg/dL)	159.33 ± 3.53	158.41 ± 3.28	NS	165.59 ± 7.99	156.27 ± 3.17	NS	NS/NS
High-Density lipoprotein (HDL) (mg/dL)	45.22 ± 0.96	40.51 ± 0.96	<0.05	49.97 ± 3.37 *	41.91 ± 0.98	<0.01	<0.05/NS
Low-Density lipoprotein (LDL) (mg/dL)	94.73 ± 3.52	90.99 ± 2.94	NS	93.56 ± 4.51	89.97 ± 2.98	NS	NS/NS
Lipoprotein Lp(a) (mg/dL)	14.24 ± 2.61	17.12 ± 2.60	NS	14.10 ± 3.21	19.70 ± 3.81	NS	NS/NS
Triglycerides (TG) (mg/dL)	97.72 ± 4.46	138.34 ± 7.59	<0.01	104.26 ± 6.98	126.88 ± 9.53	ΝA	NS/NS
Apolipoprotein (ApoA1) (mg/dL)	134.95 ± 2.14	125.45 ± 1.88	<0.01	136.06 ± 2.79	126.82 ± 2.04	<0.01	NS/NS
Apolipoprotein (ApoB) (mg/dL)	86.73 ± 2.60	89.03 ± 2.35	NS	86.46 ± 3.46 *	85.50 ± 2.36 *	NS	<0.05/<0.05
Glucose (mg/dL)	87.97 ± 1.06	86.81 ± 1.18	NS	88.79 ± 1.00	90.99 ± 0.78 *	NS	NS/<0.01
Insulin (μUI/mL)	23.65 ± 1.26	32.63 ± 1.68	<0.01	21.38 ± 1.20	31.53 ± 1.92	<0.01	NS/NS
HbA1C%	5.28 ± 0.03	5.31 ± 0.02	NS	5.23 ± 0.08	5.28 ± 0.03	NS	NS/NS
Homa-IR	5.16 ± 0.31	6.91 ± 0.38	<0.01	4.70 ± 0.26	7.11 ± 0.44	<0.01	NS/NS

All results are presented as mean ± SE. Subjects were classified as having obesity according to IOTF criteria and as having obesity with MetS according to IDF criteria at initial assessment. The table presents the comparisons between the two groups at both initial and annual assessment. All measured variables were compared by employing repeated measures ANOVA. Significant main effects were revealed in the LSD post hoc test. Statistical significance was set at (*p* < 0.05), while strong significance (*p* < 0.01) is also noted. NS: non-significant (*p* > 0.05) difference. * indicates significant difference between initial and annual assessment time-points, respectively. The *p*-values between the two time-points refer to subjects with obesity and MetS and obesity without MetS.

**Table 3:** Non-traditional cardiovascular risk factors in all subjects at initial and annual assessments.

Non-traditional CVD Risk Factors	Initial Assessment (*n* = 149)			Annual Assessment (*n* = 109)			P_Between Time-Points_
	Obesity without MetS	Obesity with MetS	P_within Baseline_	Obesity without MetS at Initial Assessment	Obesity with MetS at Initial Assessment	P_within Follow-Up_	
Triglycerides/HDL	2.26 ± 0.14	3.74 ± 0.25	<0.01	2.25 ± 0.18	3.34 ± 0.32 *	<0.05	NS/<0.05
ApoB/ApoA1	0.65 ± 0.02	0.72 ± 0.02	NS	0.64 ± 0.03	0.69 ± 0.02 *	NS	NS/<0.01
High-sensitivity C-Reactive Protein (hs-CRP, mg/L)	3.92 ± 0.66	4.23 ± 0.62	NS	3.35 ± 0.53	3.94 ± 0.61	NS	NS/NS
Adiponectin (μg/mL)	18.37 ± 2.18	20.68 ± 1.73	NS	14.48 ± 1.28 *	12.00 ± 0.84 *	NS	<0.01/<0.01
Homocysteine μmoL/L	9.90 ± 0.38	10.66 ± 0.31	NS	10.13 ± 0.41	11.38 ± 0.41	NS	NS/NS
Leptin (ng/mL)	62.28 ± 5.45	74.11 ± 4.17	NS	49.34 ± 3.96 *	43.89 ± 2.89 *	NS	<0.05/<0.01
Interleukin IL-2 (pg/mL)	7.71 ± 1.82	17.83 ± 1.96	<0.05	12.21 ± 2.41	16.82 ± 2.27	NS	NS/NS
Interleukin IL-4 (pg/mL)	2.65 ± 0.58	3.64 ± 0.42	NS	2.62 ± 1.12	3.77 ± 0.77	NS	NS/NS
Interleukin IL-6 (pg/mL)	13.75 ± 1.28	26.56 ± 1.54	<0.01	9.38 ± 1.55 *	18.71 ± 1.71 *	<0.01	<0.05/<0.01
Interleukin IL-10 (pg/mL)	8.73 ± 1.20	11.50 ± 1.06	NS	7.07 ± 1.29	12.25 ± 1.28	<0.05	NS/NS
Interleukin IL-17A (pg/mL)	7.55 ± 1.88	23.51 ± 2.86	<0.01	5.67 ± 1.52	19.21 ± 4.58	<0.05	NS/NS
Tumor Necrosis Factor (TNF) pg/mL	15.86 ± 2.39	23.89 ± 2.59	NS	10.65 ± 1.90	21.34 ± 3.55	<0.05	NS/NS
Interferon-γ (INF-γ) pg/mL	14.21 ± 1.72	21.19 ± 1.79	<0.01	10.84 ± 1.51	17.72 ± 1.51	<0.05	NS/NS

All results are presented as mean ± SE. Subjects were classified as having obesity according to IOTF criteria and as having obesity with MetS according to IDF criteria at initial assessment. The table presents the comparisons between the two groups at both initial and annual assessment. All measured variables were compared by employing repeated measures ANOVA. Significant main effects were revealed in the LSD post hoc test. Statistical significance was set at (P < 0.05), while strong significance (*p* < 0.01) is also noted. NS: non-significant (*p* > 0.05) difference. * indicates significant difference between initial and annual assessment time-points, respectively. The *p*-values between the two time-points refer to subjects with obesity and MetS and obesity without MetS.

**Table 4:** Ultrasonography findings (G) in all subjects at initial and annual assessments.

Echocardiography and Ultrasonography	Initial Assessment (*n* = 149)			Annual Assessment (*n* = 109)			P_Between Time-Points_
	Obesity without MetS	Obesity with MetS	P_within Baseline_	Obesity without MetS at Initial Assessment	Obesity with MetS at Initial Assessment	P_within Follow-Up_	
Doppler 2D ECHO IVSd (mm)	8.01 ± 0.21	9.60 ± 1.01	NS	7.98 ± 0.21	8.63 ± 0.17	NS	NS/NS
Doppler 2D ECHO IVSs (mm)	8.47 ± 0.25	9.67 ± 0.30	<0.01	8.58 ± 0.24	9.63 ± 0.28	<0.05	NS/NS
Doppler 2D ECHOLVIDd (mm)	44.56 ± 0.73	46.84 ± 0.44	NS	46.62 ± 0.83	47.00 ± 0.63	NS	NS/NS
Doppler 2D ECHO LVIDs (mm)	26.23 ± 0.89	28.67 ± 0.56	NS	28.48 ± 0.82	29.17 ± 0.54	NS	NS/NS
Ejection Fraction EF (%)	66.30 ± 0.92	67.11 ± 0.66	NS	63.04 ± 1.32	67.06 ± 0.87	NS	NS/NS
Carotid Ultrasound Right Common Carotid Artery RCCA (mm)	0.50 ± 0.02	0.65 ± 0.02	<0.01	0.47 ± 0.02	0.55 ± 0.02 *	NS	NS/<0.01
Carotid Ultrasound Left Common Carotid Artery LCCA (mm)	0.50 ± 0.02	0.65 ± 0.03	<0.01	0.48 ± 0.02	0.57 ± 0.03 *	NS	NS/<0.01
Mean Common Carotid Artery intima-media thickness c-IMT (mm)	0.50 ± 0.02	0.65 ± 0.03	<0.01	0.48 ± 0.02	0.56 ± 0.02 *	NS	NS/<0.01

All results are presented as mean ± SE. Subjects were classified as having obesity according to IOTF criteria and as having obesity with MetS according to IDF criteria at initial assessment. The table presents the comparisons between the two groups at both initial and annual assessment. All measured variables were compared by employing repeated measures ANOVA. Significant main effects were revealed in the LSD post hoc test. Statistical significance was set at (*p* < 0.05), while strong significance (*p* < 0.01) is also noted. NS: non-significant (*p* > 0.05) difference. * indicates significant difference between initial and annual assessment time-points, respectively. The *p*-values between the two time-points refer to subjects with obesity and MetS and obesity without MetS.

**Table 5:** Presence or absence of NAFLD in the subjects with MetS and without MetS at initial and annual assessment.

	Initial Assessment		*p* Value	Annual Assessment		*p* Value
	Obesity without MetS	Obesity with MetS		Obesity without MetS	Obesity with MetS	
NAFLD	35 (25.3%)	71 (51.5%)	<0.05	21 (25%)	41 (48.8%)	NS
No NAFLD	18 (13.1%)	14 (10.1%)	NS	13 (15.5%)	9 (10.7%)	NS

Comparisons between groups were performed using a chi-square test. NS, non-significant difference.

**Table 6:** Association of NAFLD with cIMT, IL-6, and HOMA-IR index.

	Initial Assessment	Annual Assessment	P_Between Time Points_
	cIMT(mm)t_0_	cIMT(mm)t_12_	
NAFLD	0.60 ± 0.02	0.54 ± 0.02 *	<0.01
No NAFLD	0.56 ± 0.03	0.49 ± 0.03	NS
	IL-6 t_0_	IL-6 t_12_	
NAFLD	22.59 ± 1.44	15.21 ± 1.44 *	<0.01
No NAFLD	16.94 ± 1.99	15.12 ± 3.19 *	<0.05
	HOMA-IR t_0_	HOMA-IR t_12_	
NAFLD	6.49 ± 0.33	6.44 ± 0.38	NS
No NAFLD	5.65 ± 0.56	5.52 ± 0.62	NS

All measured variables were compared using repeated measures ANOVA. * indicates a statistically significant difference between baseline and annual assessment time-points. NS, non-significant difference (*p* > 0.05).

**Table 7:** Best predictors of carotid intima-media thickness.

Independent Variables	Dependent Variable (b)	*p* Value
Anthropometric parameters at initial assessment (Wt, Ht, BMI, waist and hip circumference, WHR, WHtR)		
Height 0′	cIMT 0′ (b = 0.284) cIMT 12′ (b = 0.271)	*p* < 0.05
Metabolic syndrome parameters at initial assessment (SBP, WC, glucose concentration, TG, HDL)		
ΝA		
Glucose metabolism and insulin sensitivity parameters at initial assessment (glucose, insulin, HbA1C, HOMA-IR)		
HOMA-IR 0′	cIMT 0′ (b = 0.365)	*p* < 0.05
Adiposity parameters at initial assessment (adiponectin and leptin concentrations, WC, WHtR, IL-6)		
IL-6 0′	cIMT 0′ (b = 0.254) cIMT 12′ (b = 0.441)	

## Data Availability

The data presented in this study are available on request from the corresponding author. The data are not publicly available due to privacy restrictions.

## References

[1] Benjamin E.J., Virani S.S., Callaway C.W., Chamberlain A.M., Chang A.R., Cheng S., Chiuve S.E., Cushman M., Delling F.N., Deo R. (2018). Heart Disease and Stroke Statistics-2018 Update: A Report From the American Heart Association. Circulation.

[2] Berenson G.S., Srinivasan S.R., Bao WBerenson G.S., Srinivasan S.R., Bao W. (1997). Precursors of cardiovascular risk in young adults from a biracial (black-white) population: The Bogalusa Heart Study. Ann. N. Y. Acad. Sci..

[3] Strong J.P., Malcom G.T., McMahan C.A., Tracy R.E., Newman W.P., Herderick E.E., Cornhill J.F. (1999). Prevalence and extent of atherosclerosis in adolescents and young adults: Implications for prevention from the Pathobiological Determinants of Atherosclerosis in Youth Study. JAMA.

[4] McGill HCJr McMahan C.A., Herderick E.E., Malcom G.T., Tracy R.E., Strong J. (2000). Origin of atherosclerosis in childhood and adolescence. Am. J. Clin. Nutr..

[5] Valle M., Martos R., Gascón F., Cañete R., Zafra M.A., Morales R. (2005). Low-grade systemic inflammation, hypoadiponectinemia and a high concentration of leptin are present in very young obese children, and correlate with metabolic syndrome. Diabetes Metab..

[6] Desideri G., De Simone M., Iughetti L., Rosato T., Iezzi M.L., Marinucci M.C., Cofini V., Croce G., Passacquale G., Necozione S. (2005). Early activation of vascular endothelial cells and platelets in obese children. J. Clin. Endocrinol. Metab..

[7] Lamprokostopoulou A., Moschonis G., Manios Y., Critselis E., Nicolaides N.C., Stefa A., Koniari E., Gagos S., Charmandari E. (2019). Childhood obesity and leucocyte telomere length. Eur. J. Clin. Investig..

[8] Toupance S., Karampatsou S.I., Labat C., Genitsaridi S.M., Tragomalou A., Kassari P., Soulis G., Hollander A., Charmandari E., Benetos A. (2022). Longitudinal Association of Telomere Dynamics with Obesity and Metabolic Disorders in Young Children. Nutrients.

[9] Alberti K.G., Eckel R.H., Grundy S.M., Zimmet P.Z., Cleeman J.I., Donato K.A., Fruchart J.C., James W.P., Loria C.M., Smith S.C. (2009). Harmonizing the metabolic syndrome: A joint interim statement of the international diabetes federation task force on epidemiology and prevention; national heart, lung, and blood institute; American heart association; world heart federation; international atherosclerosis society; and international association for the study of obesity. Circulation.

[10] Esser N., Legrand-Poels S., Piette J., Scheen A.J., Paquot N. (2014). Inflammation as a link between obesity, metabolic syndrome and type 2 diabetes. Diabetes Res. Clin. Pract..

[11] Heymsfield S.B., Wadden T.A. (2017). Mechanisms, pathophysiology, and management of obesity. N. Engl. J. Med..

[12] Miller J.M., Kaylor M.B., Johannsson M., Bay C., Churilla J.R. (2014). Prevalence of metabolic syndrome and individual criterion in US adolescents: 2001-2010 National Health and Nutrition Survey. Metab. Syndr. Relat. Disord..

[13] Sperling L.S., Mechanick J.I., Neeland I.J., Herrick C.J., Després J.P., Ndumele C.E., Vijayaraghavan K., Handelsman Y., Puckrein G.A., Araneta M.R.G. (2015). The cardiometabolic health alliance working toward a new care model for the metabolic syndrome. Cardiology.

[14] Zimmet P., Alberti K.G., Kaufman F., Tajima N., Silink M., Arslanian S., IDF Consensus Group (2007). The metabolic syndrome in children and adolescents—An IDF consensus. The metabolic syndrome in children and adolescents—An IDF consensus report. Pediatr. Diabetes.

[15] Wang Y., Ma X.L., Lau W.B. (2017). Cardiovascular Adiponectin Resistance: The Critical Role of Adiponectin Receptor Modification. Trends Endocrinol. Metab..

[16] Walldius G., Jungner I., Aastveit A.H., Holme I., Furberg C.D., Sniderman A.D. (2004). The apoB/apoA-I ratio is better than the cholesterol ratios to estimate the balance between plasma proatherogenic and antiatherogenic lipoproteins and to predict coronary risk. Clin. Chem. Lab. Med..

[17] Buturak A., Değirmencioğlu A., Bayrak F., Kırış T., Karakurt H., Demir A.R., Sürgit Ö., Ertürk M. (2016). Elective percutaneous coronary intervention leads to significant changes in serum resistin, leptin, and adiponectin levels regardless of periprocedural myocardial injury: An observational study. Anatol. J.Cardiol..

[18] Cheung C.Y., Hui E.Y., Cheung B.M., Woo Y.C., Xu A., Fong C.H., Ong K.L., Yeung C.Y., Janus E.D., Tse H.F. (2014). Adiponectin gene variants and the risk of coronary heart disease: A 16-year longitudinal study. Eur. J. Endocrinol..

[19] Blüher M., Fasshauer M., Tönjes A., Kratzsch J., Schön M.R., Paschke R. (2005). Association of interleukin-6, C-reactiveprotein, IL-10 & adiponectin plasma concentrations with measures of obesity, insulin sensitivity and glucose metabolism. Exp. Clin. Endocrinol. Diabetes.

[20] Genitsaridi S.M., Giannios C., Karampatsou S., Papageorgiou I., Papadopoulos G., Farakla I., Koui E., Georgiou A., Romas S., Terzioglou E. (2020). A Comprehensive Multidisciplinary Management Plan Is Effective in Reducing the Prevalence of Overweight and Obesity in Childhood and Adolescence. Horm. Res. Paediatr..

[21] Karampatsou S.I., Genitsaridi S.M., Michos A., Kourkouni E., Kourlaba G., Kassari P., Manios Y., Charmandari E. (2021). The Effect of a Life-Style Intervention Program of Diet and Exercise on Irisin and FGF-21 Concentrations in Children and Adolescents with Overweight and Obesity. Nutrients.

[22] Paltoglou G., Raftopoulou C., Nicolaides N., Genitsaridi S.M., Karampatsou S.I., Papadopoulou M., Kassari P., Charmandari E. (2021). A Comprehensive, Multidisciplinary, Personalized, Lifestyle Intervention Program Is Associated with Increased Leukocyte Telomere Length in Children and Adolescents with Overweight and Obesity. Nutrients.

[23] Kassari P., Papaioannou P., Billiris A., Karanikas H., Eleftheriou S., Thireos E., Manios Y., Chrousos G.P., Charmandari E. (2018). Electronic registry for the management of childhood obesity in Greece. Eur. J. Clin. Investig..

[24] Tragomalou A., Moschonis G., Kassari P., Papageorgiou I., Genitsaridi S.M., Karampatsou S., Manios Y., Charmandari E. (2020). A National e-Health Program for the Prevention and Management of Overweight and Obesity in Childhood and Adolescence in Greece. Nutrients.

[25] World Health Organisation (WHO) (2008). Waist Circumference and Waist–Hip Ratio: Report of a WHO Expert Consultation, Geneva, 8–11 December 2008. World Health Organ.

[26] Conway J.M., Ingwersen L.A., Moshfegh A.J. (2004). Accuracy of dietary recall using the USDA fivestep multiple-pass method in men: An observational validation study. J. Am. Diet. Assoc..

[27] Paruthi S., Brooks L.J., Ambrosio C., Hall W.A., Kotagal S., Lloyd R.M., Malow B.A., Maski K., Nichols C., Quan S.F. (2016). Consensus Statement of the American Academy of Sleep Medicine on the Recommended Amount of Sleep for Healthy Children: Methodology and Discussion. J. Clin. Sleep Med..

[28] Kastorini C.-M., Critselis E., Zota D., Coritsidis A., Nagarajan M., Papadimitriou E., Belogianni K., Benetou V., Linos A. (2019). Greek National Dietary Guidelines Scientific Team National Dietary Guidelines of Greece for children and adolescents: A tool for promoting healthy eating habits. Public. Health Nutr..

[29] Stavrou S., Nicolaides N.C., Papageorgiou I., Papadopoulou P., Terzioglou E., Chrousos G.P., Darviri C., Charmandari E. (2016). The effectiveness of a stress-management intervention program in the management of overweight and obesity in childhood and adolescence. J. Mol. Biochem..

[30] Moschonis G., Michalopoulou M., Tsoutsoulopoulou K., Vlachopapadopoulou E., Michalacos S., Charmandari E., Chrousos G.P., Manios Y. (2019). Assessment of the Effectiveness of a Computerised Decision-Support Tool for Health Professionals for the Prevention and Treatment of Childhood Obesity. Results from a Randomised Controlled Trial. Nutrients.

[31] Birmpilis A.I., Karachaliou C.E., Samara P., Ioannou K., Selemenakis P., Kostopoulos I.V., Kavrochorianou N., Kalbacher H., Livaniou E., Haralambous S. (2019). Antitumor reactive T-cell responses are enhanced in Vivo by DAMP prothymosin alpha and its C-terminal decapeptide. Cancers.

[32] Bozkurt B., Aguilar D., Deswal A., Dunbar S.B., Francis G.S., Horwich T., Yancy C. (2016). Contributory risk and management of comorbidities of hypertension, obesity, diabetes mellitus, hyperlipidemia and metabolic syndrome in chronic heart failure: A scientific statement from the American Heart Association. Circulation.

[33] Flynn J.T., Daniels S.R., Hayman L.L., Maahs D.M., McCrindle B.W., Mitsnefes M., Zachariah J.P., Urbina E.M., On Behalf of the American Heart Association (2014). Atherosclerosis, Hypertension and Obesity in Youth Committee of the Council on Cardiovascular Disease in the Young (2014) Update: Ambulatory Blood Pressure Monitoring in Children and Adolescents: A Scientific Statement From the American Heart Association. Hypertension.

[34] McLaughlin T., Reaven G., Abbasi F., Lamendola C., Saad M., Waters D., Simon J., Krauss R.M. (2005). Is there a simple way to identify insulin-resistant individuals atincreased risk of cardiovascular disease?. Am. J. Cardiol..

[35] Grover S.A., Levington C., Paquet S. (1999). Identifying adults at low risk for significant hyperlipidemia: A validated clinical index. J. Clin. Epidemiol..

[36] Musso C., Graffigna M., Soutelο J., Honfi M., Ledesma L., Miksztowicz V., Pazos M., Migliano M., Schreier L.E., Berg G.A. (2011). Cardiometabolic risk factors as apolipoprotein B, triglyceride/HDL-cholesterol ratio and C-reactive protein, in adolescents with and without obesity: Cross-sectional study in middle class suburban children. Pediatr. Diabetes.

[37] Sellers E.A., Singh G.R., Sayers S.M. (2009). Apo-B/AI ratio identifies cardiovascular risk in childhood: The Australian Aboriginal Birth Cohort study. Diabetes Vasc. Dis. Res..

[38] Okamoto M., Ohara-Imaizumi M., Kubota N., Hashimoto S., Eto K., Kanno T., Kubota T., Wakui M., Nagai R., Noda M. (2008). Adiponectin induces insulin secretion in vitro and in vivo at a low glucose concentration. Diabetologia.

[39] Rabe K., Lehrke M., Parhofer K.G., Broedl U.C. (2008). Adipokines and insulin resistance. Mol. Med..

[40] Hidekatsu Y., Hiroshi Y. (2019). Beneficial Effects of Adiponectin on Glucose and Lipid Metabolism and Atherosclerotic Progression: Mechanisms and Perspectives. Int. J. Mol. Sci..

[41] Bittencourt C., Piveta V.M., Oliveira C.S., Crispim F., Meira D., Saddi-Rosa P., Giuffrida F., Reis A.F. (2014). Association of classical risk factors and coronary artery disease in type 2 diabetic patients submitted to coronary angiography. Diabetol. Metab. Syndr..

[42] Javier A., Magaña G., Daniela M.-M., Carla E., Angulo Rojo G., de la Peña D. (2020). Association of Total and High Molecular Weight Adiponectin with Components of Metabolic Syndrome in Mexican Children. J. Clin. Res. Peadiatric Endocrinol..

[43] Francisco V., Pino J., Campos-Cabaleiro V., Ruiz-Fernández C., Mera A., Gonzalez-Gay M.A., Gualillo O. (2018). Obesity, Fat Mass and Immune System: Role for Leptin. Front. Physiol..

[44] Ghadge A.A., Khaire A.A. (2019). Leptin as a predictive marker for metabolic syndrome. Cytokine.

[45] Madeira I., Bordallo M.A., Rodrigues N.C., Carvalho C., Gazolla F., Collett-Solberg P., Medeiros Cl Bordallo A.P., Borges M., Monteiro C., Ribeiro R. (2017). Leptin as a predictor of metabolic syndrome in prepupertal children. Arch. Endocrinol. Metab..

[46] Ge L., Linxin X., Yanglu Z., Lujiao L., Junling F., Qian Z., Naishi L., Xinhua X., Changhong L., Jie M. (2017). Leptin-adiponectin imbalance as a marker of metabolic syndrome among Chinese childrenand adolescents: The BCAMS study. PLoS ONE.

[47] Budak N., Yazici C., Oztürk A., Bayram F., Mazicioğlu M.M., Kurtoglu S. (2009). Is plasma homocysteine level associated with metabolic syndrome components in adolescents?. Metab. Syndr. Relat. Disord..

[48] Soriano-Guille L., Hernandez-Garcı B., Pita J., Domınguez-Garrido N., Del Rıo-Camacho G., Rovira A. (2008). High-sensitivity C-reactive protein is a good marker of cardiovascular risk in obese children and adolescents. Eur. Endocrinol..

[49] Kim J.H., Lim J.S. (2022). The association between C-reactive protein, metabolic syndrome, and prediabetes in Korean children and adolescents. Ann. Pediatr. Endocrinol. Metabol..

[50] Paltoglou G., Schoina M., Valsamakis G., Salakos N., Avloniti A., Chatzinikolaou A., Margeli A., Skevaki C., Papagianni M., Kanaka-Gantenbein C. (2017). Interrelations among the adipocytokines leptin and adiponectin, oxidative stress and aseptic inflammation markers in pre- andearly-pubertal normal-weight and obese boys. Sci. Bus. Media N. Y..

[51] Roberts J., Fallon P., Hams E. (2019). The Pivotal Role of Macrophages in Metabolic Distress. Macrophage Activation-Biology and Disease.

[52] Pozza R., Ehringer-Schetitska D., Fritsch P., Jokinen E., Petropoulos A., Oberhoffer R. (2015). Intima media thickness measurement in children: A statement from the Association for European Paediatric Cardiology (AEPC) Working Group on Cardiovascular Prevention endorsed by the Association for European Paediatric Cardiology. Atherosclerosis.

[53] Stein J.H., Korcarz C.E., Hurst R.T., Lonn E., Kendall C.B., Mohler E.R., Najjar S.S., Rembold C.M., Post W.S. (2008). Use of carotid ultrasound to identify subclinical vascular disease and evaluate cardiovascular disease risk: A consensus statement from the American Society of Echocardiography Carotid Intima-Media Thickness Task Force endorsed by the Society for Vascular Medicine. J. Am. Soc. Echocardiogr..

[54] Younossi Z.M. (2019). Non-alcoholic fatty liver disease—A global public health perspective. J. Hepatol..

[55] Pacifico L., Perla F.M., Roggini M., Andreoli G., D’Avanzo M., Chiesa C. (2019). A Systematic Review of NAFLD-Associated Extrahepatic Disorders in Youths. J. Clin. Med..

